# The Applications and Features of Liquid Chromatography-Mass Spectrometry in the Analysis of Traditional Chinese Medicine

**DOI:** 10.1155/2016/3837270

**Published:** 2016-11-10

**Authors:** Bingyao Pang, Ying Zhu, Longqing Lu, Fangbing Gu, Hailong Chen

**Affiliations:** ^1^Department of Infectious Diseases, The First Affiliated Hospital of Dalian Medical University, Dalian, Liaoning, China; ^2^Department of General Surgery, The First Affiliated Hospital of Dalian Medical University, Dalian, Liaoning, China

## Abstract

With increasingly improved separation of complex samples and detection of unknown material capabilities, liquid chromatography coupled with mass spectrometry (LC-MS) has been widely used in traditional Chinese medicine (TCM) research. This article describes the principles of liquid chromatography (LC) and mass spectrometry (MS) and their advantages and disadvantages in qualitative and quantitative analysis of TCM. We retrieved research literatures about the application of LC-MS in TCM published during the past five years at home and abroad. To better guide the analysis of TCM, this review mainly focuses on the applications category of LC-MS, how often different kinds of LC-MS are used, and the qualitative and quantitative ability of various LC-MS in the study of TCM.

## 1. Introduction

 Thousands of years of accumulation of experience on life and disease made by our ancestors finally translated into a modern pharmacy. Chinese herbs, with complex and various ingredients, are usually put into practice by prescription, following the rules of monarch, minister, assistant, and guide. When applied, the amount of single herbs and pharmaceutical formulations can vary, which may lead to changes in the interaction between the drugs and the active ingredient. In different drug application sites, there will be differences in ingredients. Because of the complexity of the chemical composition of TCM, different chemical compositions may also react with each other. Although the practical application has proved the effectiveness of TCM, it is still difficult to explain the specific drug active ingredients of TCM in modern science. In recent years, a compound's in vivo metabolism and mechanism of action have become a hot Chinese medicine research topic. But its characteristics, such as complex composition, mechanism of multitarget and multimode of action, and role of ingredients, gives the research of TCM a certain degree of difficulty.

LC-MS fully integrates the high separation capacity of the sample of LC for complex samples and the strong qualitative ability of MS [1]. Because of its high sensitivity and selectivity, LC-MS technique has been widely used in TCM research [2].

## 2. LC

Chromatography separates the mixture using the differences of the distribution coefficient between the two phases (mobile and stationary phase). According to the state of the mobile phase, chromatography can be divided into gas chromatography, liquid chromatography, and supercritical fluid chromatography, while, according to the geometric forms of the stationary phase, chromatography can be divided into column chromatography, paper chromatography, and thin layer chromatography. The most commonly used LC method is column chromatography which regards liquid as a mobile phase. High performance liquid chromatography (HPLC) is modified based on the classic liquid column chromatography.

The application of LC is divided into two categories. One of them is qualitative or quantitative for a particular composition. Qualitation is managed according to the consistency between the sample and the target component in the peak time [38]. Quantitation is performed according to the standard curve generated after standards are injected at different concentration levels. The other one is a fingerprint [39] which refers to the notion that, after the fingerprint sample has been disposed of in some way, we can obtain chromatogram or spectrogram labeled chemical characteristics by using certain methods of analysis.

LC has a great advantage on the capability of separating complex samples, so it is the most effective option when applied to separate mixtures, but not suitable to obtain structural information of the material [2, 40–48]. Qualitation finished by the contrast between the peak positions of unknown compounds and the standards is not available for monitoring of unknown compounds.

## 3. MS

Mass spectrometry is widely used in the field of TCM research due to its high selectivity, high sensitivity, and capability of providing information including relative molecular mass and structural characteristics. MS completes the qualitation using molecular mass and relevant structural information and completes quantitation by the relationships of the peak and compound content which the peak represented. Atmospheric pressure ionization (API) of MS has electrospray ionization (ESI) and atmospheric pressure chemical ionization (APCI) [49]. For many types of compounds, ESI has high sensitivity. Compared with ESI, APCI is suitable for the less polar compounds and the analysis of volatile compounds. Depending on the differences among mass analyzers used, common MS concludes quadrupole mass spectrum (Q-MS), time-of-flight mass spectrum (TOF-MS), and ion trap mass spectrometry (IT-MS) [50–53].

Tandem mass spectrometry refers to two or more MS working together. The most commonly used tandem mass spectrometry is triple-quadrupole mass spectrometry (QQQ-MS). In order to use quadrupole to conduct multistage mass spectrometry, three quadrupoles are sequentially placed, which is triple quadrupole [2]. Another type of tandem mass spectrometry, such as quadrupole-time-of-flight mass spectrometry (Q-TOF-MS) and quadrupole-ion trap tandem mass spectrometry (Q-IT-MS), also consists of a variety of quality analyzer series [54–58]. Ion trap time series can achieve multistage MS scans sequentially at different times, so this study categorized IT-MS as tandem mass spectrometer.

Tandem mass spectrometry can induce fragments of molecular ions generated by first-stage MS, according to which we can infer the relationship between child and parent, obtain structural information of the molecule and then suggest the structure of the compound, and conduct the qualitation analysis for known and unknown compounds more accurately.

Although MS can provide structural information of a material, it requires higher purity for the sample. In TCM research, it is generally used in combination with LC.

## 4. LC-MS

LC-MS technique, using LC as a separation system and MS as a detection system, finally achieves the spectrum. When the LC and MS work together, they can carry out multistage MS to speculate the structure of the compound, thus finishing qualitative and quantitative analysis more accurately [1].

Retrieving the papers on LC-MS in the application of TCM research literatures published during the past five years at home and abroad, we found that they could be separated into two categories, that is, LC-Q-MS and LC-MS/MS. In order to analyze the differences and the advantages and disadvantages of each method, papers were classified according to the difference of tandem mass spectrometry.

### 4.1. LC-Q-MS

LC-Q-MS can be used to conduct qualitative and quantitative analysis for standard components, as shown in Table 1. When conducting qualitative analysis, we can obtain structural information through scanning positive or negative ions [3]. We can also make it clear which kind of substance the chemical structure belongs to by comparing it with the standard literatures [3, 4]. The LC-Q-MS quantifies compounds based on the selection of specific ions for monitoring [5]. In the absence of standards, LC-Q-MS can conduct the qualitative analysis for the substance using strong qualitative features of mass spectrometry [3].

LC-Q-MS can analyze substances qualitatively, but some isomers or identical molecular weight substances need to be further explored, whose structures require being further identified by reference to multistage mass spectrometry. Moreover, inaccurate qualitation leads to bias of quantitation.

### 4.2. LC-MS/MS

#### 4.2.1. LC-IT-MS

IT-MS captures different quality range of ion making use of different sizes of RF voltage applied to the ring electrodes. It has high sensitivity, and a single IT-MS can achieve multilevel mass spectrometry functions [6, 7].

IT-MS can realize time-series multistage MS scans in different chronological orders, providing ingredient MSn fragmentation; thus, it is suitable for qualitative matter, providing structural information for the identification of unknown composition, as shown in Table 2. In the study of TCM, LC-IT-MS is commonly used in qualitative composition [6–11].

#### 4.2.2. LC-QQQ-MS

Triple quadrupole means that three quadrupoles are orderly placed. Each quadrupole has a separate function. Scan modes of triple quadrupole include full-scan mode, product ion scan mode, parent ion scan mode, neutral loss scan mode, selected ion scan mode, and multiple reactions monitoring scan mode [12, 13].

LC-QQQ-MS is mainly used for quantification [12–24], as shown in Table 3. Some studies are used for qualitation [25, 26]. Compared with LC-Q-MS, LC-QQQ-MS can select particular ions to collide and analyze the fragments after collision. LC-QQQ-MS can detect the parent ion and daughter ion at the same time, so it is accurate, sensitive, and comprehensive. LC-QQQ-MS can be applied to a wider range. Compared with other tandem mass spectrometries, triple quadrupole has the best quantitative reproducibility. LC-QQQ-MS is the most commonly known substance quantitative method.

#### 4.2.3. LC-Q-TOF-MS

LC-Q-TOF-MS is generally used for qualitation. It has higher detection sensitivity and mass resolution. LC-Q-TOF-MS can accurately measure mass [59].

LC-Q-TOF-MS is mainly used for material qualitative analysis [27–37], as shown in Table 4. Compared with several other tandem mass spectrometries, it has advantages in detection sensitivity, mass accuracy, and resolution. High resolution and mass accuracy give LC-Q-TOF-MS better qualitative capability for fragment ions. It is also more persuasive when distinguishing ions' structure and isomers besides parsing unknown structures in this method [37].

## 5. Conclusions

Compared with LC-MS and LC-MS/MS, the research of LC-MS/MS is conducted relatively more, which has a higher accuracy of qualitative analysis for known and unknown compounds. LC-IT-MS, because of its capability of multiple levels of mass spectrometry, has a better performance when assessing an unknown composition structure. LC-Q-IT-MS has a better quality of resolution than LC-IT-MS. Compared with the other three kinds of LC-MS/MS, LC-QQQ-MS was found to be more often applied for research, with a better quantitative ability than others. Its qualitative capability is good, but not as good as LC-Q-TOF-MS yet. LC-Q-TOF-MS is mainly used for qualitative analysis of matter. Some of its abilities are relatively excellent, such as the sensitivity for detection, mass accuracy, and resolution.

Qualitative methods are different among different LC-MS methods, while the quantitative methods are basically the same, because all quantitative methods require standard with different concentrations. Quantitative methods are usually divided into two different types: the external standard method and the internal standard method. External standard method means that, to conduct the quantitative analysis, a standard curve is generated firstly whose horizontal axis represents concentrations and vertical axis represents the color peak spectra area. Then, according to the peak area, qualitative analysis can be done. Internal standard method means the internal standard with known concentration is added to the solutions of sample and hybrid reference substance firstly; then, a standard curve is generated whose horizontal axis represents the ratio of reference sample concentration to internal standard sample concentration and vertical axis represents the ratio of standard peak area to internal standard peak area. Lastly, the internal standard sample is injected. According to the peak area of test indicators and the internal standard, qualitative analysis can be done.

In the research of TCM, it is concluded that LC-Q-TOF-MS is a better choice in qualitative analysis while LC-QQQ-MS proves to be better for quantitative analysis. Quantitative analysis is performed using specific standard with specific concentration. Qualitative analysis can measure the structure of some unknown ingredients and speculate their attributions according to information provided by existing research literatures and database. Standard validation is the most accurate for qualitative material. Because of the complex composition and unknown abilities of TCM, research methods relatively cannot meet the need yet. Further research and exploration are still necessary.

## Figures and Tables

**Table 1:** LC-Q-MS research literatures in the application of traditional Chinese medicine published during the past five years at home and abroad.

Standards	Traditional Chinese medicine	Qualitative	Quantitative	Ref.
5-HT and NE	Rhodioloside	+	+	[3]
Calycosin-7-O-*β*-D-glucoside, and so forth	Tou nong san	+	−	[4]
Salvianolic acid D	Salvianolic acid D	+	+	[5]

**Table 2:** LC-IT-MS research literatures in the application of traditional Chinese medicine published during the past five years at home and abroad.

Standards	Traditional Chinese medicine	Qualitative	Quantitative	Ref.
Danshensu and so forth	Jitai tablets	+	−	[6]
Baicalin and so forth	Huangqin tang	+	−	[7]
—	Shu-yu capsule	+	−	[8]
Ginsenoside Rg1 and so forth	Si-ni decoction analogous formulae	+	−	[9]
Ganoderic acid A and so forth	*Ganoderma lucidum*	+	−	[10]
Protocatechuic acid and so forth	Danmu injection	+	−	[11]

**Table 3:** LC-QQQ-MS research literatures in the application of traditional Chinese medicine published during the past five years at home and abroad.

Standards	Traditional Chinese medicine	Qualitative	Quantitative	Ref.
Ginsenosides Rc and CK	*Panax *genus plants	+	+	[12]
Protopine and so forth	Jitai tablet	+	+	[13]
Rutin R1 and so forth	*Actinidia valvata *leaves	+	+	[14]
Neobavaisoflavone and so forth	*Psoralea corylifolia *L.	+	+	[15]
—	*Eclipta prostrata *L.	+	+	[16]
Naringin and so forth	Chaihu-shu-gan-san	+	+	[17]
Psoralen and so forth	Bushen zhuanggu formula	+	+	[18]
Berberine and so forth	Huang lian jie du decoction	+	+	[19]
Fuziline and so forth	Mahuang-fuzi-xixin decoction	+	+	[20]
Rutin	*Ginkgo biloba *L.	+	+	[21]
Liquiritin and so forth	Gan-sui-ban-xia decoction plus-minus gansui and gancao drug combination	+	+	[22]
(−)-Epigallocatechin and so forth	*Ginkgo biloba *leaves	+	+	[23]
Pedunculoside and so forth	*Ilex rotunda*	+	+	[24]
Gallic acid and so forth	Guizhi fuling wan	+	−	[25]
Matrine and so forth	Yandureqing	+	−	[26]

**Table 4:** LC-Q-TOF-MS research literatures in the application of traditional Chinese medicine published during the past five years at home and abroad.

Standards	Traditional Chinese medicine	Qualitative	Quantitative	Ref.
Fuziline and so forth	Mahuang-fuzi-xixin decoction	+	−	[20]
Catalpol and so forth	Jieduquyuziyin prescription	+	−	[27]
Chrysoeriol-7-O-glucoside and so forth	*Lonicerae macranthoides*	+	−	[28]
Amygdalin and so forth	San-ao-tang	+	−	[29]
Nicotinate and so forth	Saikosaponins	+	−	[30]
Betaine and so forth	Hu-gan-kang-yuan capsules	+	−	[31]
Chlorogenic acid and so forth	Yinhuang drop pill	+	+	[32]
—	Bufeiyishen formula	+	−	[33]
Ferulic acid and so forth	Rhizoma Chuanxiong	+	−	[34]
Ononin and so forth	Guge fengtong tablet	+	−	[35]
Columbamine and so forth	Roots of* Coptis chinensis *Franch.	+	−	[36]
Grosvenorine I and so forth	*Siraitiae fructus*	+	+	[37]
